# Enhancing Work-Focused Supports for People with Severe Mental Illnesses in Australia

**DOI:** 10.1155/2012/863203

**Published:** 2012-07-01

**Authors:** Natalia Contreras, Susan L. Rossell, David J. Castle, Ellie Fossey, Dea Morgan, Caroline Crosse, Carol Harvey

**Affiliations:** ^1^Monash Alfred Psychiatry Research Centre, The Alfred and Monash University, Central Clinical School, Melbourne, VIC 3004, Australia; ^2^Brain and Psychological Sciences Research Centre, Swinburne University of Technology, Burwood Road, Hawthorn, VIC 3122, Australia; ^3^The University of Melbourne and St. Vincent's Hospital, Melbourne, VIC 3065, Australia; ^4^Department of Occupational Therapy, School of Allied Health, La Trobe University, Bundoora, VIC 3086, Australia; ^5^Psychosocial Research Centre, Department of Psychiatry, University of Melbourne, 130 Bell Street, Coburg, VIC 3058, Australia; ^6^Social Firms Australia (SoFA), 10/49 Smith Street, Fitzroy, VIC 3065, Australia; ^7^North West Area Mental Health Service, 130 Bell Street, Coburg, VIC 3058, Australia

## Abstract

Persons with severe mental illness (SMI) have reduced workforce participation, which leads to significant economic and social disadvantage. This theoretical review introduces the strategies that have been implemented to address this issue. These include Individual Placement and Support (IPS) services, the most widely researched form of supported employment, to which cognitive remediation has more recently been recognised in the USA, as an intervention to improve employment outcomes by addressing the cognitive impairments often experienced by people with SMI. The authors review the international literature and discuss specifically the Australian context. They suggest that Australia is in a prime position to engage clients in such a dual intervention, having had recent success with increasing access to supported employment programs and workforce reentry, through implementation of the Health Optimisation Program for Employment (HOPE). Such programs assist with *gaining* and *maintaining* employment. However, they do not address the cognitive issues that often *prevent* persons with SMI from effectively participating in work. Thus, optimising current interventions, with work-focused cognitive skills development is critical to enhancing employment rates that remain low for persons with SMI.

## 1. Introduction

High unemployment amongst people with severe mental illness (SMI) has become an area of much concern in the mental health and public policy [1, 2] sectors alike. Numerous studies have concluded that employment status is highly correlated with social, economic, and health outcomes and overall quality of life. From a personal perspective, employment promotes a sense of purpose, self-esteem, independence and greater satisfaction with finances [3, 4]. It provides daily routine, social involvement, and personal achievement [5] and provides opportunities to affirm one's ability and feel useful to others [6, 7]. Stable employment has also been associated with a decreased level of risk for exacerbation of psychiatric symptoms [8, 9] and less frequent substance abuse [10]. It is thus not surprising that the vast majority of individuals experiencing SMI express a desire to work and consider it as a key aspect of their life [11, 12]. Despite the clear benefits of a paid vocation for this subgroup of the community, unemployment rates in people with SMI worldwide are high. In the UK, unemployment rates for this group are estimated to be between 61%–73%. This rises as high as 75%–85% in the United States [13]. In Australia, workforce nonparticipation rates for people with SMI are estimated to be between 70–78%, with many not having access to vocational rehabilitation or support to gain (and importantly, maintain) employment [13]. Of particular concern, workforce non-participation rates in Australia have remained stable despite low national unemployment rates and an increased policy focus on promoting employment opportunities for people with SMI [14].

Many aspects of life are contingent upon the income and relationships sustained by paid employment [6]. In addition to the financial ramifications, lack of work has negative effects on people's health, daily routines, sense of worth, social inclusion, and civil participation. Prolonged unemployment serves to worsen the constant strains imposed by poverty, isolation, and loss of self-respect and identity [15, 16]. There are enormous challenges in enabling individuals experiencing SMI to access employment and, once gained, helping them to sustain their positions. In spite of the existence of some comprehensive supported employment programs, a great deal of more research needs to be completed to better understand and improve their efficacy.

Much of the research in this area, reviewed below, has emanated from the United States and the UK; there is a paucity of empirical literature on employment outcomes for people with SMI in an Australian context. It will thus be of critical importance for future research to investigate the validity of these current international research conclusions in Australian settings.

## 2. From a Prevocational Training Model to an Individual Placement and Support Model

Historically, prevocational training, transitional employment and supported employment, programs have been the principal approaches to assist persons with SMI to commence or return to work [17]. Traditionally, prevocational training programs aimed to involve clients in work-related activities on the assumption that training and preparation are the first steps to achieving a permanent and paid job. This approach assumed that participants required training in a less demanding and supervised environment prior to attempting paid employment [18].

In the 1950s, a small group of clients and social work volunteers met outside of the ward of a mental hospital to provide a place of social support, activities, and introducing patients to vocational environments [19, 20]. As with other prevocational trainings, this “Clubhouse model” was based on the principle that all persons with SMI could accomplish work-related needs by promoting their autonomy in an integrated environment [17]. Clients were encouraged to participate in two arms of preparation. Firstly, a prevocational “work-ordered day” included assisting with basic daily activities (e.g., kitchen duties, welcoming others or visiting patients at the hospital), working side by side with members of staff and taking responsibility for maintaining the clubhouse [20]. Secondly, a “transitional employment program” provided participants with part-time and time-limited job placements, with supervision and minimum salary. The ultimate goal was to help clients acclimatise to the work environment, develop job skills and increase their confidence before entering the competitive job market [18, 21].

Fountain House, located in New York, is considered the pioneer of clubhouse-style employment support for persons with an SMI. Its stated aim was to place clients with SMI in work situations as a primary way of overcoming employment-related barriers and discrimination. Its approach also established strategies and principles that over the years have broadened into other eclectic vocational services. Despite these, good quality research has concluded that the Fountain House model had relatively modest outcomes in returning participants to work in real life settings. For instance, international surveys (ICCD 1996 Clubhouse Survey (USA)) reported that 19.6% of clients from all 173 clubhouses located in the United States succeeded in working in transitional employment, whereas 17.5% of members participated in independent competitive employment [22]. Extensive prevocational training, the lack of contact with real work settings, lack of continuity in the same job and consideration of employers' preferences over employees' needs have been weaknesses associated with the pre-vocational model [17].

“Supported employment” (SE) is a widely recognised evidence-based practise (EBP), developed as an alternative approach to increasing persons with an SMI capacity to contribute to the workforce. SE is focused on providing clients with assistance to enter the mainstream workforce [23], operating as part of a formal mental health service or as an independent agency. Internationally, SE programs take many forms including Individual Placement and Support (IPS) services, the open employment model and others [24]. Of these, IPS is the most extensively researched model and has demonstrated showing superior competitive employment outcomes in comparison to other models [25]. The IPS model, established by Robert Becker and Deborah Drake in the 1990s, is based on seven evidenced-based principles:

- supported employment services focus on helping clients to achieve competitive jobs,
- eligibility is based on client choice,
- rapid job search,
- integration of mental health and employment services,
- attention to clients' preferences,
- individualized job supports, and,
- personalized benefits counseling [23, 26].

The IPS Fidelity Scale has been developed to ensure that these principles and procedures are replicated with precision and reliability across different sites [23]. The scale is a 15-item tool developed in 1995 by Gary Bond and his colleagues to evaluate services' quality and feedback areas for improvement to their practices [27]. Many authors have proposed that the incorporation of fidelity and quality control measures might improve vocational outcomes and enhance the probability of IPS' success [23, 27–29].

In contrast with the pre-vocational and transitional employment models outlined above, IPS has been shown to reduce the length of the prevocational training phase [18], encouraging participants to learn new skills in real work settings, as well as keeping participants active, motivated, and engaged in job-seeking. An essential key for this model has become the integration of an “employment specialist” into the mental health service who will support the client to find a job matched to his or her preferences and provides support to the employee once in the work setting [30]. Ideally, employment specialists are integrated into the client's treatment team, so as to facilitate communication between the mental health service, client, and employer [31, 32]. Thus, the role of the employment or vocational specialist is considered a crucial feature in supported employment services, working in collaboration with clinical specialists as part of a comprehensive multidisciplinary and holistic treatment approach [33]. The employment specialist's success will be determined partially by their expertise in communicating with members of the clinical team, external employment agencies, potential employers, and government support services. But also, it will depend on negotiating effective supports with clients and people's disclosure issues, for instance, if clients want health information shared with employers. For participants who have experienced severe mental health issues, IPS emerges as meaningful and proactive support in which vocational goals and non-vocational domains like self-esteem, control of psychiatric symptoms and quality of life are positively affected [34].

Abundant evidence has indicated the effectiveness of supported employment programs (particularly IPS) in helping people with SMI to achieve employment in the last two decades. Many literature reviews [9, 35, 36], systematic reviews [26, 37–40], more than twelve randomized controlled trials [23, 41, 42] and longitudinal follow-up studies [43, 44], albeit mostly in the United States and Europe, have demonstrated that supported employment provides sufficient tools and support to clients to obtain jobs, with benefits persisting over the years [44, 45]. Further, it has been shown that IPS offers considerably more positive outcomes than a variety of other traditional models [46], including psychosocial rehabilitation programs [47] and sheltered workshops [34, 48, 49]. For instance, studies find that people participating in IPS programs earn higher wages, work more hours per month, have lower attrition rates [31, 37, 47] and less frequent visits to mental health services [43] than participants in comparison programs.

These positive aspects of the IPS model are well recognised, but research has consistently shown that the benefits of IPS in assisting participants to maintain their employment for lengthy periods are modest [26, 50]. 

## 3. Job Tenure

Brief job tenure continues to be a problematic issue for workers with SMI. Despite supported employment showing improvements in general vocational performance in people with SMI and higher competitive employment rates than prevocational approaches (50%–70% compared with 35%) [34, 46, 50, 51], several studies confirm the low percentage of participants who experience sustained benefit from it, with the majority of workers experiencing brief job tenure and unsuccessful job endings [52–54]. Empirical research has shown that, on average, clients with SMI maintain their jobs for six months [18, 48, 55]. Yet there is great variation, with some clients remaining employed for a longer time (9–11 months) [34, 56] and others lasting only a few months or weeks [31, 47].

The high rates of job termination in clients with SMI pose questions regarding the efficacy of the IPS model. Supporting people to sustain employment has proven more challenging than job acquisition. Significant factors contributing to the failure to support sustained employment are postulated to be insufficient attention to the lifestyle and attitude adjustments that may be required to adapt to working life; lack of specific knowledge, strategies and supports for managing mental illness, and sources of stress within and beyond the workplace; financial barriers, poor matching of jobs to worker interests [6, 57, 58] and lack of specific skills in managing cognitive impairments that are directly associated with poor vocational outcomes [59].

People with SMI who find employment that matches with their job preferences are more likely to stay in their jobs for longer [52, 54]. Thus, motivated and satisfied participants employed in positions that fulfil their personal preferences have been shown to stay in their jobs twice as long as participants in roles that do not personally interest them [54]. Higher sense of self-confidence, on-the-job permanent support, professional qualifications [56], and cognitive performance [60] have been other variables predictive of better vocational performance and longer job tenure.

## 4. A Supported Employment Program in Australia

In Australia, the National Mental Health and Disability Employment Strategy [61] forms a significant part of the government's social inclusion agenda. It acknowledges that for people living with a disability, exclusion from employment and social participation persists even as the country enjoys its lowest unemployment rate ever in a time of significant economic growth and labour shortages. During the past decade, the Australian Federal Government (composed of six states and two territories) has focused its mental health reforms on the promotion of key services. These include increasing evidence-based treatments and the improvement of integrative practices between employment and mental health services. Nonprofit, nongovernmental organizations have also contributed to these reforms through the development of new employment and educational policies, providing major social inclusion for clients participating in these various settings [62]. 

Since 2006 the supported employment model (also referred to as Open Employment [63]) has been implemented in Australia with comparative employment results within the international sphere. Consistent with research conducted in other comparable Western countries, Australian and New Zealand studies have reported employment rates for people with SMI between 46% and 65% [64–66]. In spite of these positive outcomes, the system still presents structural weaknesses leading to diminished quality and outcomes of the overall service. The incorporation of vocational assistance into the public mental health service still remains as the central challenge for the mental health plan [31, 62, 67]. In 2006, seven pioneers within mental health provided support employment programs to job seekers with severe mental illness located in four different states in Australia. Subsequent studies have shown that according to each site's report, the main difficulties that professionals encountered were the time taken to incorporate the “vocational specialist” into the mental health program, the lack of training for both the specialists and the rest of the mental health team, lack of resources to accomplish the specialist's requirements, and organizational culture differences [62]. 

These observations are comparable with the results of studies done worldwide. For example, in England between January 2010 and March 2011, nine sites implementing the IPS model (some of them more experienced than others) participated in a fidelity research as part of a national programme undertaken by the Centre for Mental Health. Consequently, and based on 16 IPS fidelity reviews, Shepherd and colleagues (2012) concluded that some of the system's weaknesses and, therefore, areas to be improved were related to the process of integration of the Supported Specialist into the clinical team; the phenomenon of resistance, cultural differences, and professional prejudices; the process of obtaining referrals and the importance of a good quality training for supervisors [68]. 

The findings demonstrate the need for further policy and program development to establish effective and durable relationships between employment and health providers, firstly to enhance the overall IPS programs' outcomes and secondly to enable improved delivery to people with SMI to gain and maintain competitive employment.

## 5. LEAP-HOPE

In 2009, the LEAP (Local Employment Access Partnerships) were established by Social Firms Australia (SoFA) as part of a program funded by the Australian Department of Education, Employment, and Workplace Relations. The LEAP project was initiated to improve the delivery of services to job seekers with an SMI by enhancing the communication and collaboration between relevant support services. Clinical mental health services, psychiatric disability rehabilitation services, and disability employment services are engaged in the partnerships. These three service systems work with the same client cohort (people with SMI) and all have a role in assisting clients to achieve their vocational aspirations. However, the services have varying approaches to program delivery, different methods of measuring outcomes and are funded by different state and federal agencies, creating difficulties for collaboration. Historically, the lack of coordination between these services has reduced their ability to assist job seekers with a mental illness to secure or retain employment. 

LEAP now operates in five Melbourne locations and one regional Victorian area. A total of 43 agencies are engaged with the LEAP partnerships. 

Working through the LEAP partnerships, SoFA (Social Firms Australia) delivers the Health Optimisation Program for Employment (HOPE). HOPE is a ten-session psychoeducational group program for jobseekers with a “mental health issue” that aims to offer them a better understanding of the situations and stressors that affect their health, and to learn new strategies to manage their illness in the context of securing work. The HOPE program was adapted by SoFA and St Vincent's Health from an existing evidence-based program developed by Professor David Castle [69].

The core program—health optimisation—has been run in a variety of settings with a range of different populations over the past ten years. Evidence has shown that the program is effective in reducing symptomatology and duration of hospitalisations [69]. HOPE is delivered as a collaborative activity of the LEAP partnerships and centrally administered by SoFA. Participants are recruited through LEAP partner agencies and must be current clients to ensure that they receive support if issues arise while participating in HOPE.

To date, 198 participants have completed the program with overall positive vocational outcomes: 33% of clients who participated in the program achieved employment and an additional 34% secured volunteer work or returned to study. A statistically significant increase in self-efficacy was reported and was sustained over time. Despite its positive vocational outcomes, there are still a significant number of consumers that do not manage to return to any competitive employment after the HOPE program and sustaining employment is likely to continue to be a challenge. The inevitable question then raised is what new approaches have recently evolved to address these issues?

## 6. Thinking Skills for Work

People with SMI present a wide range of cognitive deficits; they are most pronounced in the areas of attention, memory, processing speed, and executive functions [70–74]. In many cases individuals are aware of these deficits and complain about severe difficulties in solving simple daily life problems, paying attention to social activities and responding to educational and vocational opportunities. Cognitive impairments are highly associated with poor social functioning, specifically in the capacity for reasoning, processing of social information and solving interpersonal problems [75–77]. In the last twenty years, advances in psychological interventions have explored the field of innovative rehabilitation technology, focusing on the enhancement of thinking skills as central to improved functional outcomes. 

Cognitive Remediation Therapy (CRT) is an empirically well-supported model which considered a skill-training intervention that facilitates cognitive improvements, by providing training in memory, attention, and other cognitive abilities [73, 78]. Several studies have shown that CRT provides additional psychosocial benefits for those clients who participate in comprehensive rehabilitation programs, including social skills training or supported employment [79, 80]. In recent years, Susan McGurk has developed a novel program called “Thinking Skills for Work (TSW)” that integrates the training of cognitive functions and the development of strategies to manage the persistent cognitive difficulties in real job settings with support received from an employment consultant [81]. For these purposes, the TSW includes the computer-based cognitive training CogPack 6.0 software, in addition to permanent support with job search planning, finding work and coping with daily challenges arising in the workplace. During the last decade McGurk and her colleagues have conducted a number of randomized controlled trials [76, 81, 82] and longitudinal follow-up studies [83] with positive results. Clients that received vocational training plus the cognitive remediation therapy, compared with those that only participated in the vocational rehabilitation program, reported more hours of work, higher wages, and improvements in cognitive skills, especially in verbal learning, memory, and executive functions. These studies have supported Cognitive Remediation as a reliable tool in improving cognitive and vocational functioning in participants attending TSW programs. Also, this comprehensive rehabilitation program (CRT+IPS) has been associated with significant improvements in depression and autistic preoccupation. The most relevant conclusion from this body of research for this paper, however, is the strong (but as yet unreplicated) finding that improvements in cognitive functioning predict longer job tenure and reduced job termination rates. 

## 7. Conclusion

A number of different training programs and support initiatives have been trialled to address restricted workforce participation in people with SMI and improve their vocational outcomes. Despite advances in the area, unemployment remains high, and job tenure continues to be short-lived even after intensive support. Estimates suggest that only 10–20% of mental health service clients are working in mainstream employment settings [13, 63, 84]. Clearly there is much more work to be done. 

Supported employment has shown success internationally in increasing employability, but less well developed are programs to address the cognitive impairments that likely play an important part in job sustainability in this population. A focus on cognitive remediation was largely born in response to mental health service clients' reports of struggling with severe cognitive impairments on the job. The *Thinking for Work* program offers a potential new way to address these underlying issues with the wider goal of increasing job tenure over time. This program has already been shown to be effective overseas, and it is appropriate to attempt to replicate these successes in Australia.

Much more research in this area is needed. There is still no consensus in the literature on the role or importance of motivational factors or which specific facets of cognition are relevant to vocational stability. It is for these reasons that investigating the utility of Thinking Skills for Work in an Australian context becomes a crucial area of interest.
